# Effects of low-load blood flow restriction on the venous system in comparison to traditional low-load and high-load exercises

**DOI:** 10.3389/fphys.2023.1285462

**Published:** 2023-12-15

**Authors:** Alexander Franz, Sanghyeon Ji, Frank Sebastian Fröschen, Marleen Kerstin, Patrick Wahl, Michael Behringer

**Affiliations:** ^1^ Department of Orthopedics and Trauma Surgery, University Hospital Bonn, Bonn, Germany; ^2^ Department of Adult Reconstruction, ATOS Orthoparc Clinic Cologne, Cologne, Germany; ^3^ Section Exercise Physiology, Institute of Exercise Training and Sport Informatics, German Sport University Cologne, Cologne, Germany; ^4^ German Research Centre of Elite Sport (momentum), German Sport University Cologne, Cologne, Germany; ^5^ Department of Sports Sciences, Goethe University Frankfurt, Frankfurt, Germany

**Keywords:** venous occlusion, kaatsu training, venous hypertension, venous function, resistance training, physical training

## Abstract

**Purpose:** Blood-Flow-Restriction (BFR) training provides the ability to achieve hypertrophy effects even though only light mechanical loads are applied. However, its impact on venous pressures and function are still unknown. Therefore, the present study investigates the influence of BFR-training on intravascular venous pressure and venous function in comparison to control exercises with low or high mechanical loads.

**Methods:** In a randomized cross-over design, ten healthy men (27.6 ± 6.4 years) underwent three trials of unilateral knee-extensor exercise with three different training protocols, low-load- (LL-RT, 30% of the individual 1-repetition-maximum, 1RM), low-load BFR- (LL-BFR-RT, 30% 1RM, 50% limb occlusion pressure, LOP) and high-load resistance exercise (HL-RT, 75% 1RM). Exercise protocols contain about four sets of knee extension exercise (Range-of-Motion: 0-0-95°), separated by 60 s of rest. Each set was performed until volitional muscle failure. For analysis of changes in intravascular venous pressures and venous function, a venous catheter was placed at the exercising leg before each trial. Whereas venous pressures were recorded throughout the exercise trials, phlebodynamometric investigations were performed before and after each trial. Furthermore, subjective pain perception during and after exercise was accessed by visual analogue scale. One-way ANOVA was used to assess mean differences between training protocols, while two-way repeated-measures ANOVA (rANOVA; time x condition) was performed to compare changes in measures over time among conditions. Data were given as means ± standard deviation (SD).

**Results:** In comparison to the exercise trials without venous occlusion, total workload was significantly lower in the LL-BFR-RT (LL-RT: 1745 ± 604 kg vs LL-BFR-RT: 1274 ± 237 kg vs HL-RT: 1847 ± 367 kg, *p* = 0.004) without indicating statistical differences in venous pressures during the exercise sets (interaction: *p* = 0.140) or pain perception (interaction: *p* = 0.574). Similarly, phlebodynamometric assessment of venous function (e.g. refill-time of the venous system pre-vs. post exercise trials–LL-RT: 29.7 ± 11.0 s vs 25.5 ± 9.6 s, LL-BFR-RT: 26.6 ± 13.0 s vs 27.3 ± 13.8 s, HL-RT: 25.9 ± 10.9 s vs 23.1 ± 8.2 s) revealed no time (*p* = 0.156), condition effect (*p* = 0.802) or their interactions (*p* = 0.382).

**Conclusion:** The present study is the first one describing the acute effects of LL-BFR-RT to muscle failure on venous pressures and function in comparison to a LL- and HL-RT in the lower limbs. In contrast to the existing literature, LL-BFR-RT does not elevate the venous pressures during exercise higher than a comparative exercise without BFR and does not show any adverse effects on venous function after the exercise.

## Introduction

It was previously assumed that an increase in muscle mass and strength could be achieved exclusively by training with high mechanical loads (American College, 2009). However, scientific work in recent decades repetitively demonstrates that such effects can also be achieved with low load resistance exercise (LL-RT) (Schoenfeld et al., 2017). While LL-RT requires a large number of repetitions to induce anabolic effects, an additional venous occlusion of the exercising limb through a blood pressure cuff is able to significantly reduce the necessary workload (Patterson et al., 2019; Silva et al., 2019). This training technique is called Blood-Flow-Restriction resistance training (BFR-RT).

The underlying mechanism of BFR-mediated effects on muscle mass and strength enhancements are still unknown in detail. While the externally applied tourniquet pressure supports a faster shift from aerobic to an anaerobic energy supply, resulting in a significant accumulation of metabolites in the occluded limb through local hypoxia (Miller et al., 2021), subsequent responsible physiological processes of muscle anabolism are under discussion. Commonly cited drivers of the enhancement in protein biosynthesis include BFR-induced rises in growth hormone concentration (Fujita et al., 2007), increased neuromuscular activity or tourniquet-induced elevations in hydrostatic filtration pressure with consecutive muscle swelling (Loenneke et al., 2012). In particular, the last issue is of major medical interest, as BFR-RT induces a significant venous hypertension of 60 mmHg above the venous pressure of a control condition at the upper extremities (Franz et al., 2020). Whether such an increase in pressure can be tolerated by the venous capacitance system or whether it is damaged by it is unknown.

The venous vessels in the human body have only a thin muscle layer in comparison to the arteries and are referred as capacity vessels due to their ability to store large amounts of blood. The purpose of the veins is on the one hand, the storage of blood, as well as its return to the heart. Based on their muscle weakness, the transport is supported by venous valves, which subdivide the upward blood flow and thus enable the successful work of the surrounding muscle pump. In general, the venous vessels are a rather under-researched area and thus there are no data available on how the venous pressure of the lower extremities changes under BFR-RT. Since the lower extremities are generally exposed to a higher venous pressure than the upper extremities in rest because of gravity (Tansey et al., 2019), a different response to altered blood flow conditions than the upper extremities can be suggested. Furthermore, it is unknown how the venous system of the legs is tolerating externally applied pressures in an acute or after several sessions of BFR-RT. If there is a limited tolerance to the stimulus, resulting in pathological venous reflux (Sarin et al., 1992), this should be applied as a contraindication for patients with venous insufficiency or in postoperative periods when lymphedema is present.

Therefore, the aim of the present study was to investigate the changes in intravenous pressure during lower extremity BFR-RT compared with low-load (LL-RT) and high-load resistance training (HL-RT) by a direct intravascular catheter approach. Furthermore, this invasive technique allows us to additionally examine possible acute effects of the exercise trials on venous function through phlebodynamometric measurements.

## Material and methods

### Sample size calculation

Based on our previous data (Franz et al., 2020), illustrating changes in intravascular pressure parameters during LL- and LL-BFR-RT, we assumed a moderate to high effect (*f* = 0.3) with respect to our main outcomes. To determine the required sample size, we conducted a power analysis using G*Power (version 3.1.9.7). Assuming a mean effect size (*f*) of 0.3, an alpha error (ɑ) of 0.05, and a statistical power (1–*β*) of 0.90, we found that a minimum sample size of *N* = 8 for a repeated measures design (within-between interaction, correlation level among repeated measures: 0.5, non-sphericity correction: 1.0) would be necessary. Therefore, the obtained sample size of *N* = 10 is more than adequate to test the study hypothesis.

### Subjects

Ten healthy male subjects (age: 27.6 ± 6.4 years) volunteered for this study (Table 1). To participate in the present study, subjects had to be > 18 years of age, free of acute illness, and have a negative history of previous vascular surgery of the lower extremities (e.g., bypass or stent-surgery) and previous blood disorders (e.g., sickle cell disease). Furthermore, all subjects were experienced in resistance training (>2 years), but had no prior experiences with BFR-RT. Subjects were informed about the experimental procedures and possible risks and signed an informed consent document before the investigation.

**TABLE 1:** Basic characteristics of participants.

Variable	
Age [y]	27.6 (6.4)
Height [cm]	188 (6)
Body mass [kg]	80.0 (5.9)
1RM/30% 1RM/75% 1RM [kg]	77.0 (10.7)/23.1 (3.2)/57.8 (8.0)
LOP/50% LOP [mmHg]	203 (12)/102 (6)

1RM, one-repetition maximum; LOP, limb occlusion pressure.

The study was approved by the local Ethics Committee (Trial-ID: 2015104498) and was performed according to the Declaration of Helsinki.

### Study design

**FIGURE 1:**
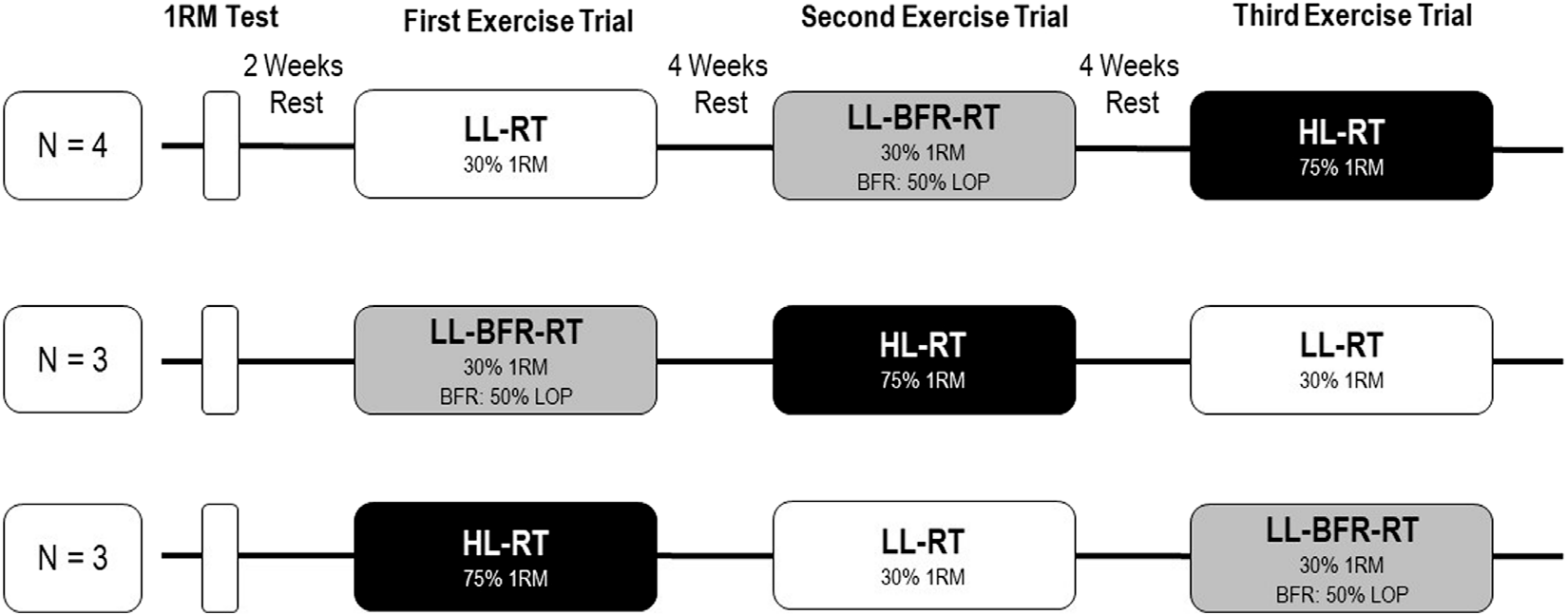
Schematic illustration of the randomized study design, containing three exercise sessions in different sequence. Abbrevations: 1RM, one-repetition-maximum; LL-RT, low-load resistance training; LL-BFR-RT, low-load blood flow restriction resistance training; HL-RT, high-load resistance training; LOP, limb occlusion pressure.

To investigate the effects of BFR-RT on venous hemostasis and function in comparison to exercise without venous occlusion to muscular failure, a randomized cross-over design was applied. Recruited subjects performed three different unilateral knee extensor exercise protocols, separated each by 4 weeks of rest. To control the impact of the repeated-bout-effect (Hyldahl et al., 2017), participants were randomized via a random-number table to start with either the LL-RT (n = 4), the LL-BFR-RT (n = 3) or the HL-RT (n = 3). Furthermore, the participants were asked to stop strength training 1 week before each experimental visit. Therefore, all subjects reported to the laboratory for four testing sessions and follow-up measurements (Figure 1).

During the first visit, subjects’ individual concentric 1-repetition-maximum (1RM) of the knee extensors of the dominant leg was determined in accordance to Jessee et al. (Jessee et al., 2017). After a 2-week rest period, participants performed the first exercise protocol at the second visit, depending on random assignment.

### Interventions

The exercise protocol consisted of unilateral leg extension exercise with the dominant leg by using a knee extensor resistance exercise machine (Compass Leg Extension, Proxomed, Alzenau, Germany) that were performed for four sets. Each set was done until voluntary muscle failure, defined as a discontinuation of the exercise due to muscle fatigue, or if the subject was not able to keep the pace, which was set to 2 s for the concentric as well as the eccentric phase, controlled by a metronome (60 beats per minute). The exercise sets were separated by 60 s of rest.

The mechanical load for the LL-RT and LL-BFR-RT trials was set to 30% of the individual 1RM. For comparison to a traditional strength training, a high-load resistance exercise trial (HL-RT) was added, which was performed without venous occlusion with a weight of 75% of the individual 1RM. Based on the performed repetitions in each set the total workload (repetitions x applied mechanical load) was determined for comparison between exercise trials.

The additional venous occlusion during LL-BFR-RT was performed by using 50% of the individual arterial limb occlusion pressure (LOP) and was hold continuously during the full four set protocol and rest phases in between. For determination of the LOP, an inflatable tourniquet of 11.5 cm width was placed proximal at the exercising leg before the training session (PBFR, Delfi medical Inc., Vancouver, Canada). After a 10-min rest period, LOP was determined automatically by the PBFR device and sonographically controlled (Lumify, Philips, Hamburg, Germany) in a lying position by displaying the femoral artery by using a Doppler to assess the blood flow within the vessel. Subsequently, the cuff was inflated until no further blood flow was detectable. This pressure was defined as the individual LOP.

### Venous pressure analysis

**FIGURE 2:**
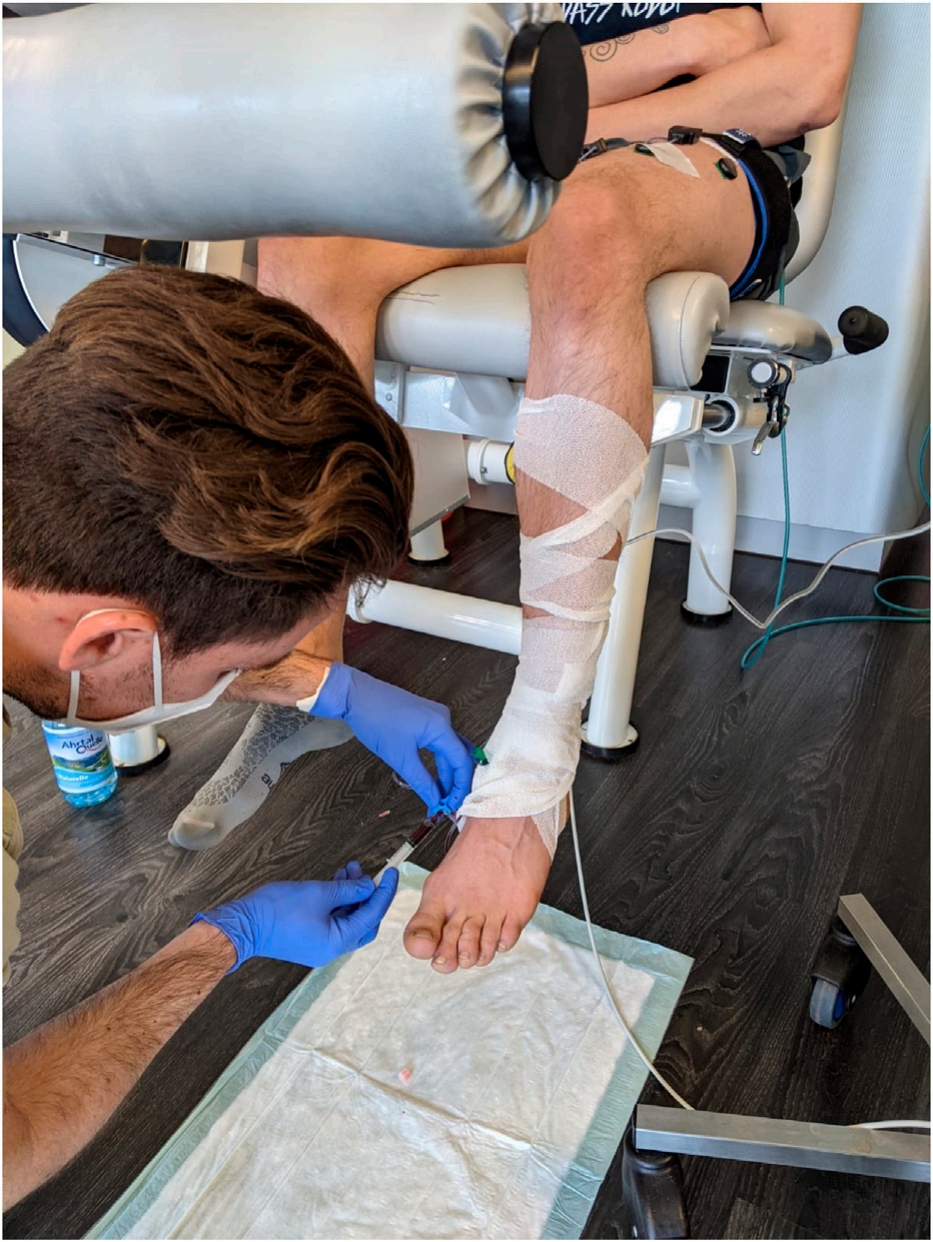
Exemplary venous sampling of a blood gas analysis on the foot before exercise.

To assess intravascular changes in venous pressure, a venous catheter was placed into the dominant leg before exercise. For this purpose, participants were placed in a lying position on a medical examination table. Under local anesthesia with lidocaine hydrochloride, a dorsal foot vein (*Rete venosum dorsale pedis*) was punctured, and a sensor needle (20 gauge) was placed into the vein which was linked to a line containing a continuous column of saline connected to a transducer unit (LogiCal Pressure Monitoring Kit, Smiths medical int. Ltd., United Kingdom) (Figure 2).

The system was calibrated outside the tissue at the patient’s heart level in a standing position. Venous pressure was recorded continuously during phelbodynamometry, throughout the exercise protocol and post-exercise measurements after placement of the venous catheter. During data collection, specific time points were selected from the continuous pressure recording to illustrate changes in intravascular pressure: before exercise, in between the four exercise sets (immediately after the end of each set), and immediately and 5 min after the exercise protocol.

### Phlebodynamometry

**FIGURE 3:**
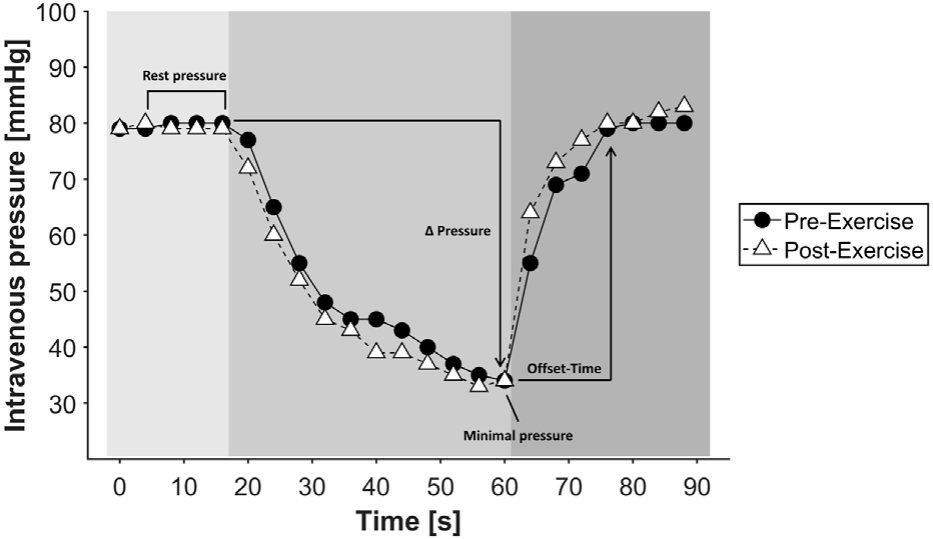
Exemplary course of a phlebodynamometry measurement of a subject before and after a low-load blood flow restriction resistance exercise trial. Light gray shading: resting phase and measurement of resting pressure in the venous system. Medium gray shading: Test phase of phlebodynamometry in which the loading protocol is performed, in this case 20 calf raises and 10 squats. Dark gray shading: recovery and refill time, refilling of the venous system.

Phlebodynamometric assessments are applied to measure venous function in the lower extremities to illustrate possible venous insufficiency. After puncture of a dorsal foot vein, a sensor needle is placed intravenously to record venous pressure at rest and during a testing protocol (Norgren and Thulesius, 1975). First, resting intravenous pressure of the subjects was measured by a 2-min rest period in a standing position. Subsequently, the subjects had to perform 20 calf raises, followed by 10 squats which is referred as the “testing protocol”. Each repetition was timed for one second by a metronome. After the testing protocol the intravenous pressure was continuously monitored for further 2 min in a resting standing position to reach baseline values. Assessed parameters were the pressures (mmHg) during rest in the standing position (rest pressure), the minimal pressure during the testing protocol (minimal pressure), the delta between the resting pressure and minimal pressure during the testing protocol (Δ pressure) and the time (s) from minimal pressure during the testing protocol to a full recovery (Offset Time), representing by the resting pressure (Figure 3).

### Subjective pain perception

Subjective pain intensity was recorded in rest, immediately after each set and after 5-, 10- and 30 min. Subjects were instructed to mark a line on a 100-mm visual analog scale (VAS) to illustrate their level of subjective discomfort and pain.

### Statistics

All statistical analyses were performed using the Statistical Package for the Social Sciences (SPSS, version 29.0, Chicago, IL, United States). Data are given as means ± standard deviation (SD), if not indicated otherwise. Normal distribution and homogeneity of variance were verified using Shapiro-Wilk and Levene’s Test, respectively. The mean differences between exercise trials were assessed using one-way ANOVA. To compare changes in measures over time among conditions, two-way repeated-measures ANOVA (rANOVA; time x condition) were performed. If the main effects for time or condition were detectable, post hoc-tests with Bonferroni correction were performed to check which factor levels differ significantly from one another. For interaction and main effects, the partial eta squared (*η*_*p*_^*2*^) was calculated as an effect size measure. According to Cohen (Cohen, 2013), a ηp^2^ ≥ 0.01 indicates small effects, ηp^2^ ≥ 0.059 medium effects, and ηp^2^ ≥ 0.138 large effects. For all results, an alpha level of 0.05 was interpreted as statistically significant.

## Results

Table 2 presents the number of repetitions performed and the total workload for each exercise trial. Total workload was significantly lower in the LL-BFR-RT compared to LL- and HL-RT (*p* < 0.05).

**TABLE 2:** Repetition of each set and total workload.

Condition	Repetitions [times]				Total workload [kg]	One-way ANOVA (p/ηp²)
	Set 1	Set 2	Set 3	Set 4		
LL-RT	28.7 (9.5)	17.6 (5.1)	14.6 (4.4)	14.2 (4.9)	1745 (604) #	0.004/0.339
LL-BFR-RT	23.6 (4.7)	12.0 (2.2)	10.6 (2.5)	9.4 (3.7)	1274 (237)	
HL-RT	11.2 (2.9)	8.0 (1.5)	6.7 (1.6)	6.4 (1.6)	1847 (367) #	

ANOVA, analysis of variance; LL-RT, low-load exercise trail; LL-BFR-RT, low-load exercise trial with blood flow restriction; HL-RT, high-load exercise trial.

*p* < 0.05, difference to LL-BFR-RT.

**FIGURE 4:**
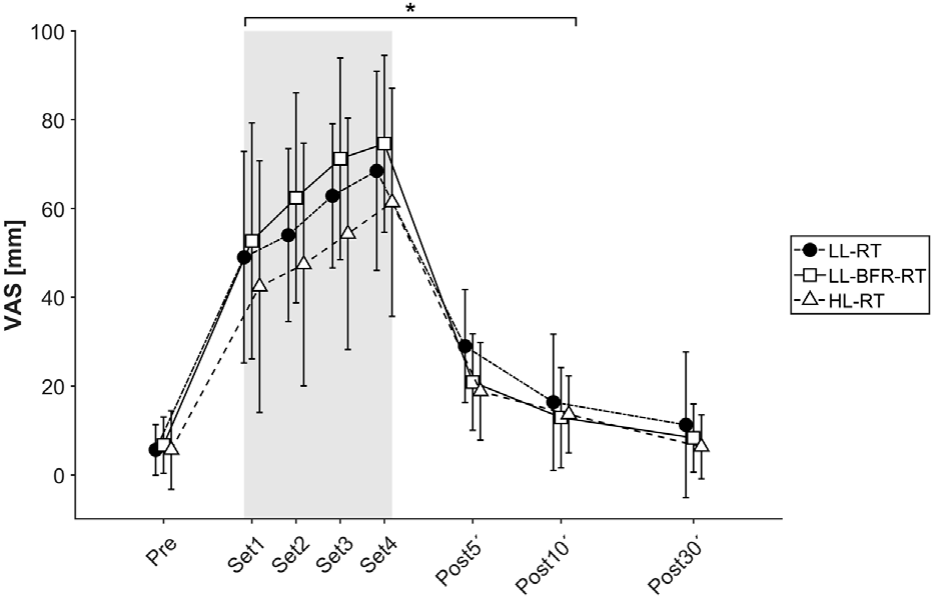
Plot of subjective pain perception measured by visual analog scale (0–100 mm) throughout the exercise trials. The course is shown as mean value with standard deviation under all three conditions: Black circle, LL-RT, low-load resistance training; white square, LL-BFR-RT, low-load blood flow restriction resistance training; white triangle, HL-RT, high-load resistance training. *Significantly different from baseline within the respective condition (*p* < 0.05).

Regarding the subjective pain perception during and after exercise accessed by VAS, we observed a significant time effect (*p* < 0.001; *η*_*p*_^*2*^ = 0.748) but no significant effects were found for interaction (*p* = 0.574; *η*_*p*_^*2*^ = 0.051) or condition (*p* = 0.434; *η*_*p*_^*2*^ = 0.065). Pain perception increased with each set of exercise and returned to the baseline level at 30 min post-exercise (see Figure 4).

**FIGURE 5:**
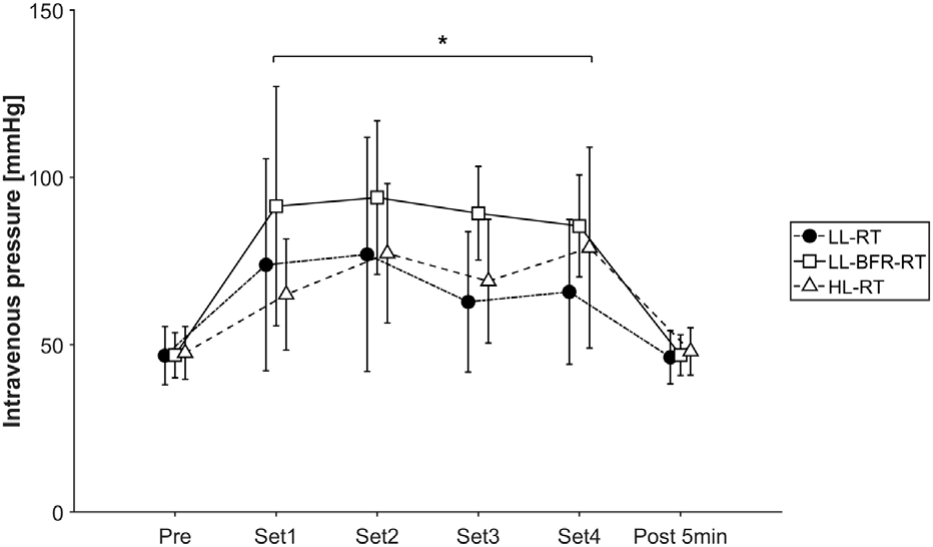
Plot of intravasal measured venous pressure (mmHg) throughout the exercise trials. The course is shown as mean value with standard deviation under all three conditions: Black circle, LL-RT, low-load resistance training; white square, LL-BFR-RT, low-load blood flow restriction resistance training; white triangle, HL-RT, high-load resistance training. *Significantly different from baseline within the respective condition (*p* < 0.05).

As shown in Figure 5, intravenous pressure increased during exercise showing significant time effect (*p* < 0.001; *η*_*p*_^*2*^ = 0.481), but there were no significant differences between conditions at any time point (interaction: *p* = 0.140, *η*_*p*_^*2*^ = 0.108; condition: *p* = 0.060, *η*_*p*_^*2*^ = 0.188).

An example of the intravenous pressure during phlebodynamometric assessment before and after the testing protocol is shown in Figure 3. We found no significant effects of any exercise trial on the measurements related to phlebodynamometry as presented in Table 3.

**TABLE 3:** Outcomes related to phlebodynamometric assessment before (Pre) and after (Post) exercise trial.

	LL-RT	LL-BFR-RT	HL-RT	rANOVA (*p/ηp²*)		
				Time	Condition	Time x condition
Rest pressure [mmHg]						
Pre	80.5 (5.0)	80.9 (8.5)	81.0 (6.6)	0.228/0.062	0.932/0.006	0.424/0.072
Post	79.6 (7.2)	79.0 (10.7)	81.3 (6.5)			
Minimal pressure [mmHg]						
Pre	34.3 (16.5)	40.7 (21.7)	40.4 (17.2)	0.121/0.097	0.797/0.019	0.554/0.048
Post	39.5 (11.6)	41.6 (21.1)	42.4 (12.2)			
Delta pressure [mmHg]						
Pre	48.6 (5.9)	45.5 (9.7)	50.1 (7.5)	0.782/0.003	0.625/0.038	0.257/0.107
Post	46.2 (8.3)	48.8 (7.6)	50.4 (10.0)			
Offset time [s]						
Pre	29.7 (11.0)	26.6 (13.0)	25.9 (10.9)	0.156/0.082	0.802/0.018	0.382/0.077
Post	25.5 (9.6)	27.3 (13.8)	23.1 (8.2)			

rANOVA, repeated-measures analysis of variance; LL-RT, low-load exercise trail; LL-BFR-RT, low-load exercise trial with blood flow restriction; HL-RT, high-load exercise trial.

## Discussion

The presented study compares for the first time intravascularly measured venous pressures during exercise trials with different mechanical loads (LL- vs. HL-RT) and LL-BFR-RT. Furthermore, this is the first study analyzing the acute effects of different exercises on venous function by phlebodynamometric assessments. However, in order to be able to classify the effects of LL-BFR-RT and the other conditions on the venous pressures, the applied exercise protocols have to be compared first regarding the necessary workload to muscular failure and subjective pain perception to identify possible differences between the trials.

### Workload and subjective pain perception

Probably the greatest benefit of BFR-RT in comparison to HL-RT is the achievement of training-induced effects on muscle strength, mass or endurance with only low mechanical loads (Kelly et al., 2020). Additionally, in comparison to LL-RT, LL-BFR-RT needs significantly less repetitions to induce necessary impulses for muscle growth in the trained muscles (Gavanda et al., 2020). Considering the assumption that muscle hypertrophy is predominantly induced by exercise to muscle fatigue (Farup et al., 2015; Dankel et al., 2017), the present data illustrate that LL-BFR-RT needs less total workload to achieve volitional muscle failure in comparison to LL- or HL-RT in four sets of knee extension. Interestingly, if LL- or HL-RT is performed to muscle failure, our data demonstrated no statistical difference in total workload between these two conditions (Table 2). Since former studies were able to show that hypertrophic effects on muscle mass and strength were similar between LL-BFR-RT and HL-RT (Grønfeldt et al., 2020), this technique could be beneficial for subjects which are not able to perform high mechanical demanding or long-lasting exercise based on reduced physical properties.

However, challenging arguments against a clinical inclusion is caused by reported higher subjective pain perception during BFR-RT in comparison to control conditions (Spitz et al., 2022). In contrast, the present data show that there were no statistical differences in pain perception between the three exercise conditions through all performed exercise sets (Figure 4). Probable reason for the conflicting results with the literature may be due to differences in the exercise protocols. The most often applied exercise protocol for LL-BFR-RT consisting of four sets with 75 repetitions (set 1: 30 reps, set 2–4: 15 reps), usually leads to muscle failure (Patterson et al., 2019; Cognetti et al., 2022). In a recent study by our research group, no subject was able to perform 75 repetitions with LL-BFR-RT in a unilateral biceps curl trial, whereas the exercise protocol without venous occlusion was completely performable and associated with less perceived pain (Franz et al., 2020). For this reason, a comparison of the BFR method to a control condition with a volume-matched protocol to describe physiological responses should be interpreted with caution. The available data show that even though the duration to exhaustion (HL-RT < LL-BFR-RT < LL-RT) and thus the necessary workload is significantly different between the investigated training forms, a load to voluntary exhaustion is not associated with different pain perception. Although no direct instrument for measuring muscular exhaustion was used (e.g., force drop), it can be summarized that the examined exercise trials leads to a similar level of subjective discomfort when performed to muscle failure.

### Venous pressure analysis during the exercise

By using intravascular venous catheters, the present study was able to record the venous pressures before, during and after the exercise sessions. In a recent study by our research group, we were able to illustrate significant elevations in venous pressure by LL-BFR-RT up to 60 mmHg in the upper extremity during unilateral biceps curls in comparison to LL-RT (Franz et al., 2020). Considering that in an upright position gravity causes a hydrostatic venous hypertension of about 35 mmHg at the hand and 90 mmHg above the ankle at rest (Arnoldi, 1965; Tansey et al., 2019), we hypothesized that BFR-RT of the lower limbs will be able to increase hypertension significantly.

Interestingly, all three exercise conditions lead to a significant increase in venous pressure, without showing a statistical group by time interaction (Figure 5). This contrary finding can be caused by several reasons. First, the result could be altered based on the positioning of the subjects during the exercise. During the standing phase, the available pressure data show resting values of about 80 mmHg, while the resting values in the sitting position are only 50 mmHg. Accordingly, the positioning of the participant during the leg extension exercise provides a significant change in resting venous pressure, causing that the illustrated effect of BFR-RT on the venous system of the lower extremities is only generalizable for seated BFR-RT exercise. Furthermore, the two studies differ based on the performed exercise protocols. While the previous exercise protocol containing an unilateral biceps curls exercise was performed with a fixed number of sets and repetitions (4 sets, 30-15-15-15 reps), the present protocol was performed up to volitional muscular failure (Franz et al., 2020). Although the BFR-RT protocol of the previous study resulted in volitional failure of the participant, this was not present in the control condition without BFR-RT. For this reason, the measured pressure data of the LL-RT condition are not comparable to those of this study, which exercised to volitional muscle failure even under control conditions.

However, even if the expected rise in venous pressure is not verifiable, it is still questionable if the venous function is negatively affected by short-term exercise-induced venous hypertension. Therefore, the present study applied a phlebodynamometric assessment before and after the exercise to evaluate venous function.

It is noteworthy that we found similar venous pressure responses between HL-RT and LL-RT. From arterial pressure measurements, it is known that HL-RT and LL-RT present similar increases, when both conditions are performed to muscular fatigue, as shown by Fleck and Dean in the late 1980s (Fleck and Dean, 1987). It is conceivable that this is also true for the venous pressure (probably as a result of arterial pressure responses), however, to date there is a lack of evidence to support this hypothesis and it warrants further investigation.

### Phlebodynamometry

In order to assess the function of the venous system and the impact of the different exercise protocols, a phlebodynamometric investigation was performed before and after each exercise trial. In a healthy venous system, exercise leads to reductions in venous pressure by acting of the muscle pump. If the exercise is stopped, the deep vein system gets refilled and a rise back to baseline values is evident. In venous insufficiency, the action of the muscle pump is not able to press the blood volume from the deep vein system upright or into the superficial venous system (Recek, 2013). This condition is mostly caused by degenerated venous valves, which normally separate the venous blood over several levels and thus support a steady upward flow of venous blood back to the heart (Sarin et al., 1992). Since BFR-RT exerts an unknown load on the venous system to which human physiology is not accustomed, BFR-RT could have adverse effects on venous function.

In the present study, subjects performed a phlebodynamometric assessment, consisting of 20 calf raises and 10 squat exercise repetitions, before and 10 min after the exercise trials. We could not detect a difference in baseline- or minimal pressures in comparison before or after, as well as between the different exercise trials. Furthermore, the present results show no difference between the refill-time of the venous system after the exercises or in the delta between resting- and minimal venous pressures. Therefore, it can be concluded that, in healthy, young males, a single exercise bout of LL-BFR-RT as well as LL- or HL-RT of the lower extremities on the leg extension machines does not affect the function of the venous system. Finally, we would like to state herewith that in the process of conducting the study, no side effects resulted from the exercise trials, interventions or measurement techniques.

While the present findings only illustrate acute effects of BFR-RT on the venous system, future studies should try to investigate venous function after repeated BFR-RT applications. Furthermore, it is questionable to what degree the results of this young, healthy and male subject cohort can be transferred to the general public. Venous insufficiencies, varicose veins or other venous pathologies occur mainly in older age. Accordingly, for a general applicability recommendation of BFR-RT also in future rehabilitative sports, a re-analysis of venous function before and after BFR-RT should be performed in elderly and possibly diseased subject groups focusing if the venous system of the aging human is also able to counteract BFR-induced short-term hypertension.

### Venous pressure response during exercise and phlebodynamometry

The observation that the venous pressure increased during the exercise protocols but dropped during Phlebodynamometry measurements, is somewhat counterintuitive. However, we believe that responses of the venous pressure differ between calf/squat and knee extensor exercise, based on the fact that the latter does not activate the calf pump. As delineated by Meissner and colleagues (Meissner, 2005), the calf pump is the most effective of all pumps with the greatest capacitance. Upon activation, it empties the veins in the posterior compartment, resulting in a pressure drop in the deep veins. This causes blood to flow from the superficial veins through the perforating veins into the deep system, which explains the observed drop in blood pressure. In the case of isolated contractions of the thigh muscles during knee extensions, this emptying of the veins in the lower leg is absent and the effect of the blood flowing in (arterial blood flow is only restricted) and the lack of outflow (venous occlusion) due to the BFR intervention dominates. However, these considerations remain hypothetical, as we do not have the tools to measure the pressures in the venous network of the knee extensors and we therefore cannot say with certainty how representative the measurement of venous pressure at the dorsum of the foot is of the pressure deeper in the tissues.

## Limitations

Our study does, however, have several potential limitations. The first limitation of our study is its low number of included participants. Nevertheless, according to the sample size calculation it is sufficient to evaluate the hypothesis. Another limitation which has to be considered is that our LOP determination for subsequent BFR-RT was done in a supine position, although the experimental exercise was performed in a sitting position. Given that the resting pressure measurement showed differences between changes in subject positioning as well, it could be that the previously measured 50% of the LOP was changed by the seating position. To what extent the influence of the cuff on the vascular system was reduced or potentiated by the position cannot be resolved by the data collected and needs clarification in future work. An additional limitation is raised by the positioning of the venous catheter. Since the intravascular assessment of venous pressure was done at the dorsal foot, it does not illustrate potential differences in pressure at the major contracting muscle during knee extension exercise. However, considering subject safety, the puncture of a deep vein on the thigh would not have been acceptable for the risk-benefit assessment of the study.

## Data Availability

The original contributions presented in the study are included in the article/Supplementary Material, further inquiries can be directed to the corresponding author.

## Supplementary material

SUPPLEMENTARY FIGURE S1 Illustration of the pressure curves during the individual sets in the three different loading protocols: Low-Load Resistance Training (LL-RT), Low-Load Blood-Flow-Restriction Resistance Training (LL-BFR-RT) and High-Load Resistance Training (HL-RT). The individual pressure curves of the subjects and a regression line (black, using the smoothing method, non-linear model) are shown. The sets are presented in relative time (%). Furthermore, the start and end pressures are shown at the beginning of the set (blue dot) and at the end of the set (red dot) as mean value and standard deviation.

SUPPLEMENTARY FIGURE S2 Illustration of the pressure curves during the individual sets in the three different loading protocols: Low-Load Resistance Training (LL-RT), Low-Load Blood-Flow-Restriction Resistance Training (LL-BFR-RT) and High-Load Resistance Training (HL-RT). The individual pressure curves of the subjects are presented in real time (s).
