# Supplementary figures and images for: Effects of low-load blood flow restriction on the venous system in comparison to traditional low-load and high-load exercises
Source: Front Physiol. 2023 Dec 15;14:1285462. doi: 10.3389/fphys.2023.1285462 (PMC10757371; doi:10.3389/fphys.2023.1285462)

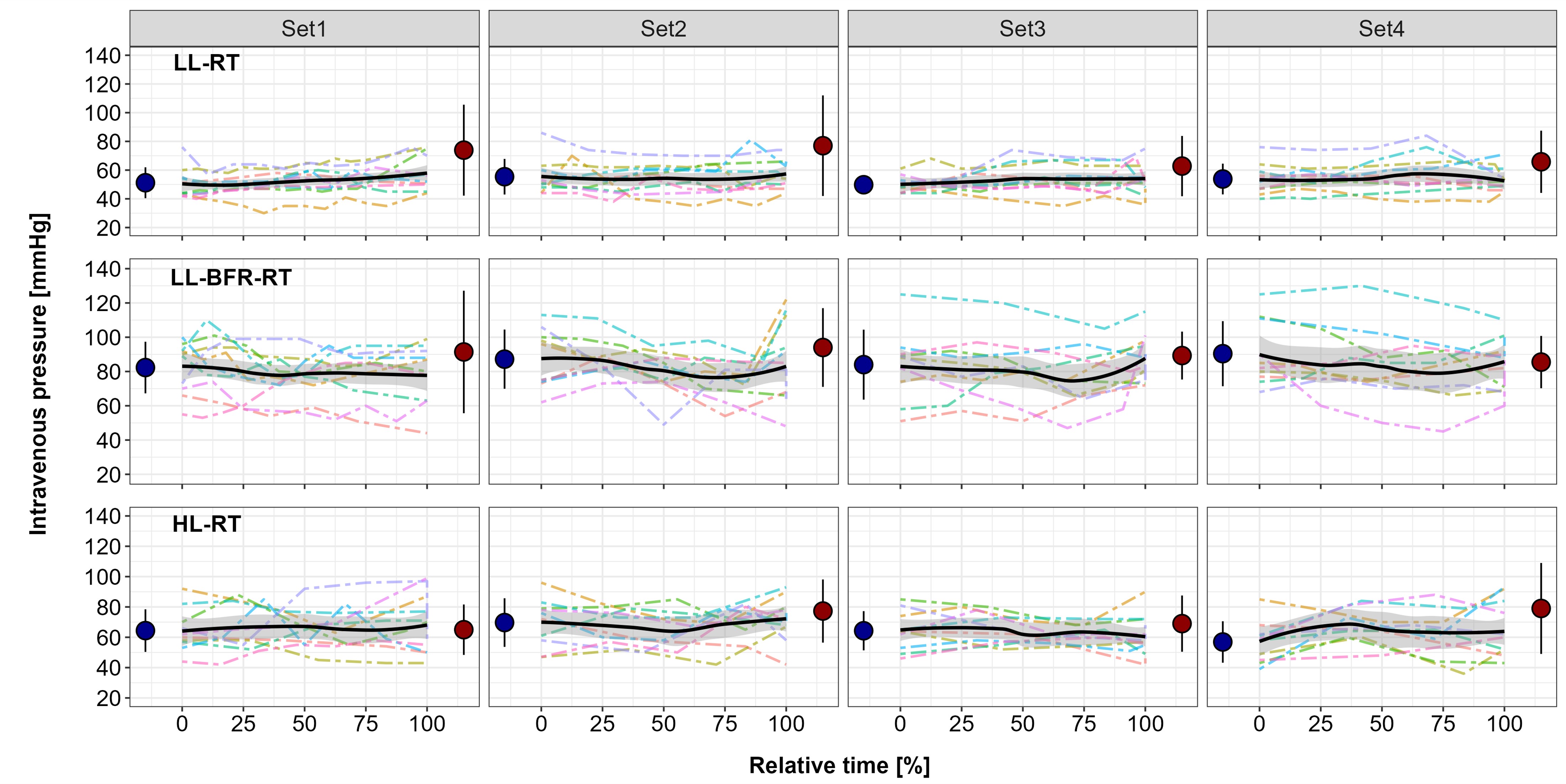

Supplement: Supplementary file 1 [file Image1.JPEG]

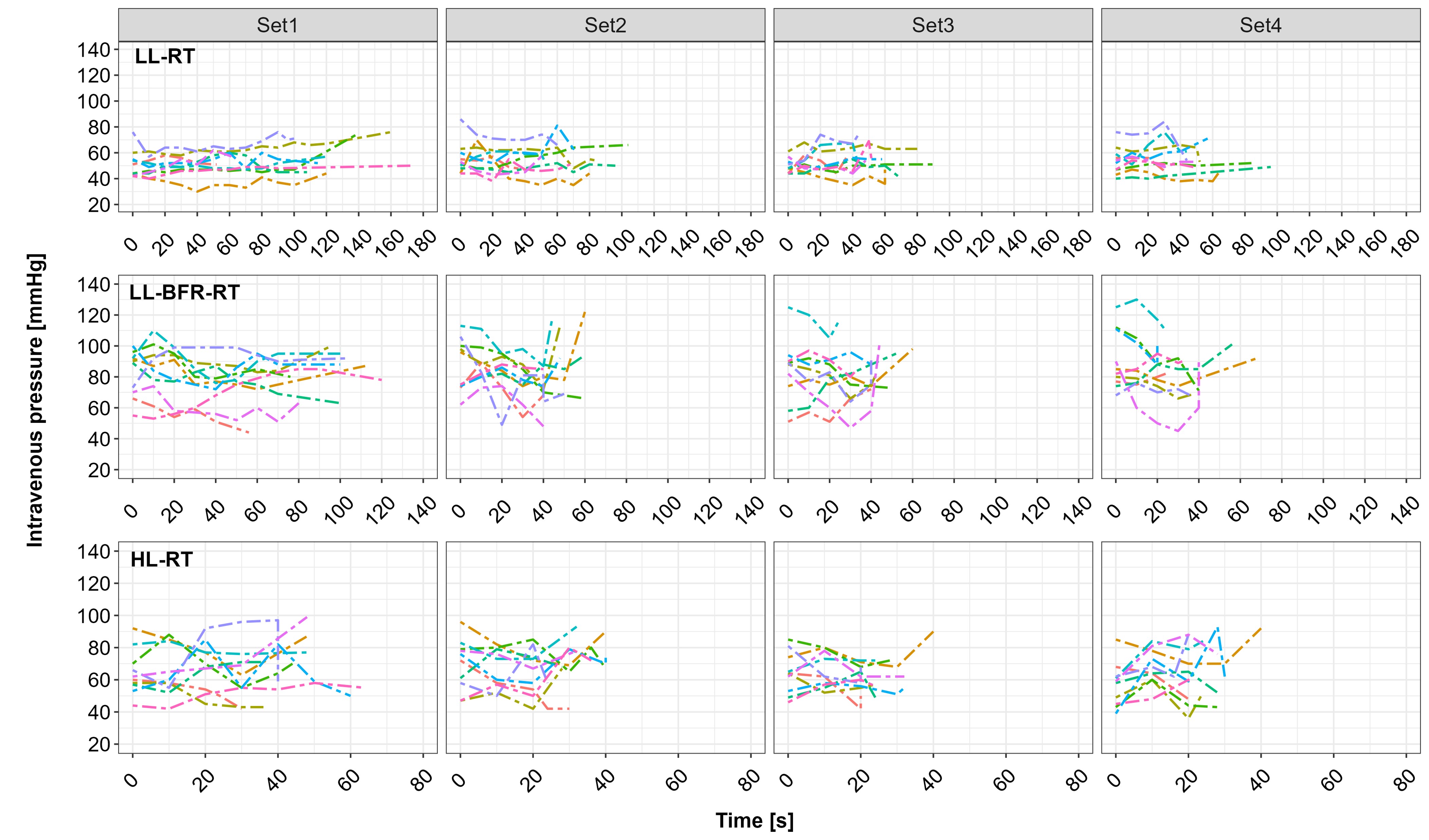

Supplement: Supplementary file 2 [file Image2.JPEG]
